# Bortezomib resistance in multiple myeloma is associated with increased serine synthesis

**DOI:** 10.1186/s40170-017-0169-9

**Published:** 2017-08-29

**Authors:** Esther A. Zaal, Wei Wu, Gerrit Jansen, Sonja Zweegman, Jacqueline Cloos, Celia R. Berkers

**Affiliations:** 1 Biomolecular Mass Spectrometry and Proteomics, Bijvoet Center for Biomolecular Research and Utrecht Institute of Pharmaceutical Sciences, Utrecht University, Padualaan 8, 3584 CH Utrecht, The Netherlands; 2 Amsterdam Rheumatology and Immunology Center—Location VUMC, VU University Medical Center, Amsterdam, The Netherlands; 3 Department of Hematology, VU University Medical Center, Amsterdam, The Netherlands; 4 Pediatric Oncology/Hematology, VU University Medical Center, Amsterdam, The Netherlands

**Keywords:** Metabolism, Drug resistance, Bortezomib, Multiple myeloma, PHGDH

## Abstract

**Background:**

The proteasome inhibitor bortezomib (BTZ) is successfully applied in the treatment of multiple myeloma, but its efficacy is restricted by the wide-spread occurrence of resistance. Metabolic alterations play an important role in cancer development and aid in the cellular adaptation to pharmacologically changed environments. Metabolic changes could therefore play an essential role in the development of drug resistance. However, specific metabolic pathways that can be targeted to improve bortezomib therapy remain unidentified.

**Methods:**

We elucidated the metabolic mechanisms underlying bortezomib resistance by using mass spectrometry-based metabolomics and proteomics on BTZ-sensitive and BTZ–resistant multiple myeloma cell lines as well as in a set of CD138+ cells obtained from multiple myeloma patients.

**Results:**

Our findings demonstrate that a rewired glucose metabolism sustains bortezomib resistance. Mechanistically, this results in higher activity of both the pentose phosphate pathway and serine synthesis pathway, ultimately leading to an increased anti-oxidant capacity of BTZ-resistant cells. Moreover, our results link both serine synthesis pathway activity and expression of 3-phosphoglycerate dehydrogenase (PHGDH), which catalyzes the rate-limiting step of serine synthesis, to bortezomib resistance across different BTZ-resistant multiple myeloma cell lines. Consistently, serine starvation enhanced the cytotoxicity of bortezomib, underscoring the importance of serine metabolism in the response to BTZ. Importantly, in CD138+ cells of clinically bortezomib refractory multiple myeloma patients, PHGDH expression was also markedly increased.

**Conclusions:**

Our findings indicate that interfering with serine metabolism may be a novel strategy to improve bortezomib therapy and identify PHGDH as a potential biomarker for BTZ resistance.

**Electronic supplementary material:**

The online version of this article (doi:10.1186/s40170-017-0169-9) contains supplementary material, which is available to authorized users.

## Background

The proteasome inhibitor bortezomib (BTZ) is widely used in the treatment of multiple myeloma (MM) [1, 2]. The proteasome is a large intracellular protease complex, composed of a 20S core particle and two 19S regulatory particles. The 20S core particle consists of two outer rings of α-subunits and two inner rings of β-subunits. Three of these β-subunits, termed β1, β2, and β5 are catalytically active [3]. They can be replaced by the three interferon inducible subunits β1i, β2i, and β5i to form immunoproteasomes. BTZ acts by inhibiting primarily the β5/β5i subunits and to a lesser extent the β1/β1i subunits [4]. The resulting imbalance between production and degradation of proteins leads to the accumulation of (regulatory) proteins, causing endoplasmic reticulum stress and activation of the unfolded protein response. Proteasome inhibition eventually causes apoptosis in malignant cells via multiple pathways, including overproduction of reactive oxygen species [5–7].

Despite good clinical results of initial treatment, many patients eventually relapse from BTZ therapy [7–9]. Resistance is associated with mutations in the binding pocket of the β5 subunit (*PSMB5*), resulting in impaired binding of the drug [10–12], upregulation of the proteasomal machinery, and a change in the ratio of (immuno) proteasomal subunits [13–15]. However, how relevant PSMB5 mutations are in relapsed patients remains unclear [16] and more recent studies suggest that other mechanisms are involved in BTZ resistance, such as the unfolded protein response and vesicular exocytosis of ubiquitinated proteins [5, 17–20].

Targeting metabolism is emerging as a promising strategy for cancer therapy [21–23]. Metabolic alterations have been linked to resistance to chemotherapeutic agents [24–27] and may also play a role in the (lack of) response to BTZ. In particular, higher glycolytic activity has been found to lower BTZ sensitivity under hypoxic conditions [28] and BTZ resistant cells were found to have proteomic changes in redox and energy metabolism [29]. However, specific metabolic targets that can serve to augment responses to BTZ therapy remain unidentified.

In this study, we aimed to identify altered metabolic pathways in BTZ-resistant MM cells that drive resistance. By using mass spectrometry and tracer-based metabolomics combined with proteomics, we show that BTZ-resistant cells adapt their glucose metabolism. Mechanistically, this metabolic rewiring results in higher activity of both the pentose phosphate pathway (PPP) and serine synthesis pathway (SSP) and ultimately leads to an increased anti-oxidant capacity of BTZ-resistant cells. We also demonstrate that serine starvation enhances the effect of BTZ and that 3-phosphoglycerate dehydrogenase (PHGDH), which catalyzes the rate limiting step in the SSP, is upregulated across different BTZ-resistant MM cells. Our results indicate that serine metabolism is associated with BTZ resistance and that interfering with this pathway may be a novel strategy to improve BTZ therapy.

## Methods

### Reagents

The proteasome activity probe Me_4_BodipyFL-Ahx_3_L_3_VS was a gift from Huib Ovaa (Leiden University Medical Center, The Netherlands). Bortezomib (BTZ) and carfilzomib (CFZ) were purchased from Selleck Chemicals. All solvents were obtained from Biosolve. All other chemicals were obtained from Sigma-Aldrich, unless stated otherwise.

### Cell culture

Human multiple myeloma RPMI-8226 wild type (WT) cells were purchased from ATCC. BTZ-resistant cells were obtained as described previously [11]. AMO-1 and ARH-77 WT, BTZ- and CFZ-resistant cells were kindly provided by C. Driessen (Kantonsspital St. Gallen, Switzerland). Cells were maintained in suspension culture in RPMI-1640 (Lonza) medium supplemented with 2 mM L-glutamine (Lonza), 10% fetal bovine serum (FBS) (Gibco) and 100 μg/ml penicillin/streptomycin (Lonza) and were kept at 37 °C in humidified 5% CO_2_ atmosphere. BTZ- and CFZ-resistant cells were continuously cultured in the presence of BTZ or CFZ as previous described [11, 13]. Cells were cultured without drugs 4–6 days prior to experiments. Media were supplemented with 10% FBS and 100 μg/ml penicillin/streptomycin unless stated otherwise.

### Proteasome activity profiling

Proteasome activity was measured using Me_4_BodipyFL-Ahx_3_L_3_VS as described previously [30]. Cells were suspended in triplicate at a density of 1 × 10^6^ cells/ml in RPMI-1640 medium and incubated with the indicated concentrations of BTZ at 37 °C for 1 h, followed by a 1-h incubation with 500 nM Me_4_BodipyFL-Ahx_3_L_3_VS. Cells were collected by centrifugation, washed with PBS and lysed for 30 min in NP40 lysis buffer (50 mM Tris, pH 7.4, 150 mM NaCl, 1% NP40) at 4 °C, followed by centrifugation at 14,000*g* to remove membrane fractions, nuclei and cell debris. Protein concentrations were determined using the Bradford assay (Bio-rad) and equal amounts of protein were denatured by boiling in XT Sample buffer (Bio-rad) with 9% β-mercaptoethanol. Proteins were separated on a 4–12% SDS-PAGE gel (Bio-rad) and fluorescence was measured with a Typhoon scanner (GE Healthcare) (λ_ex_/λ_em_ = 488/526 nm). Protein loading was confirmed with a coomassie blue stain.

### Cell viability and cell growth assays

Cells were suspended in triplicate at a density of 2–5 × 10^5^ cells/ml in RPMI-1640 medium in 96-well plates and incubated with drugs at the indicated concentrations for 24–48 h. Cell growth was monitored continuously with the IncuCyte live-cell imager system. Images were automatically acquired every 2 h for 1–2 days. Pictures were analyzed using the IncyCyte Zoom software. Cell growth was defined as the amount of cell doublings per 24/48 h and calculated based on increase of confluency. Cell death was assessed after 24–48 h by incubating each well with 30 μM propidium iodide and measuring fluorescence after 15 min using the IncuCyte live-cell imager system. Cell death was calculated based on the area of the fluorescent signal, normalized to confluency of the wells. Cell viability was measured in parallel after 24–48 h by incubation of cells with 50 μM resazurin for an additional 2 h, after which absorption was measured at 570 nm and 600 nm using a Multiskan GO microplate reader (Thermo Scientific). Results were calculated by subtraction of background absorbance at 600 nm from absorbance at 570 nm.

### Liquid chromatrography—mass spectrometry (LC-MS)-based metabolomics

For all experiments, cells were diluted in fresh medium 16–24 h prior to the start of experiments. ^13^C–tracer experiments were performed as described [31, 32], with minor changes. At the start of all experiments, cells were counted and centrifuged for 5 min at 1400 rpm to remove the old medium. Cells were then resuspended in DMEM containing 8 mM [U-^13^C]D-glucose (Cambridge Isotopes) at a density of 1 × 10^6^ cells/ml, unless indicated otherwise. After 4 or 8 h, samples were washed with PBS and harvested by centrifugation for 5 min at 1000*g* at 4 °C. At these timepoints, cells had recovered from centrifugation and reached pseudo-steady state, without nutrients being depleted from the culture media. For all analyzed metabolites, (near) isotopic steady state was reached at these time points. In addition, samples were harvested after 24 h to analyze serine levels in the cells. Because at this point some nutrients were depleted, no other metabolites were analyzed in these samples. Metabolites were extracted by adding 100–200 μl ice-cold MS lysis buffer (methanol/acetonitrile/uLCMS H_2_O (2:2:1)) to the cell pellets. To measure extracellular metabolites, medium samples were obtained prior to harvesting cells at 8 or 24 h. Metabolites were extracted by diluting 10 μl medium in 1 mL MS lysis buffer. To measure differences in extracellular metabolites in different BTZ-resistant cell lines, cells were resuspended at a density of 1 × 10^6^ cells/ml in in Minimal Essential Medium (MEM), supplemented with 1 mM L-glutamine, 0.2 mM L-serine and 0.2 mM L-glycine. Medium samples were obtained after 8 h and metabolites were extracted as described above. For serine starvation experiments, medium was formulated to match the composition of DMEM [31]. Medium consisted of MEM, supplemented with additional 1× MEM vitamins, 1× MEM amino acids, 10% dialyzed FBS and glucose up to 25 mM, in the presence or absence of 0.4 mM L-serine. Cells were resuspended at a density of 0.7 × 10^6^ in triplicate wells and were pre-incubated for 24 h in the presence or absence of serine. After 24 h, cells were centrifuged at 1400 rpm for 5 min and media was replaced with matched media containing [U-^13^C]D-glucose. After an additional 4 h, cells were harvested as described above.

Metabolites were analyzed by LC-MS (see Additional file 1: methods for details). Metabolites were identified based on exact mass within 5 ppm and further validated by concordance with retention times of standards. Peak areas of identified metabolites were in their respective linear range of detection. Metabolites were quantified using LCquan software (Thermo Scientific). Because cells were resuspended at equal cell densities, no substantial cell growth occurred between 0 and 8 h, and growth rates between resistant and sensitive cell lines were comparable up to 24 h, samples were assumed to contain equal cell numbers. Peak intensities were additionally normalized based on median peak intensity to correct for technical variations during mass spectrometry analysis. Isotopomer distributions were corrected for natural abundance and data are plotted as relative peak area compared to WT cells under control conditions.

### DHA uptake assay

Cells were suspended at a density of 0.5 × 10^6^ cells in 200 μl in MEM supplemented with 1 mM L-glutamine and 0.2 mM L-serine. Cells were incubated with 5 mM dehydroascorbic acid (DHA) for 5 min at 37 °C. DHA uptake was terminated by addition of 1.5 ml cold PBS. Cells were centrifuged at 1000*g* for 5 min at 4 °C and lysed in 75 μl MS lysis buffer. Samples were centrifuged at 16.000*g* for 15 min at 4 °C to remove precipitated proteins and cell debris and the supernatants were collected for LC-MS analysis.

### GSH/GSSG ratio

Cells were suspended at a density of 2.5 × 10^6^ cells/ml in triplicate wells of 12-well plates and incubated in the indicated conditions. Cells were washed with cold PBS and lysed in 10 mM HCl by two freeze/thaw cycles. Proteins were precipitated by addition of 1% 5-sulfosalicylic acid and pelleted by centrifugation at 4 °C for 10 min at 8000*g*. The ratio of reduced to oxidized glutathione (GSH/GSSG) in the supernatant was measured using the quantification kit for oxidized and reduced glutathione (Sigma-Aldrich) according to the manufacturer’s instructions.

### Proteomics

Cells were suspended in RPMI medium at a density of 1 × 10^6^ cells/ml and incubated for 2 h. Three replicates of 5 × 10^6^ cells were washed with PBS and centrifuged for 5 min at 4 °C. Cell pellets were lysed at room temperature by gentle vortexing in 8 M urea, 50 mM ammonium bicarbonate, 2% Triton X-100 and 0.1% SDS, supplemented with phosphatase inhibitor (PhosSTOP, Roche) and protease inhibitor (cOmplete mini EDTA-free, Roche). Total lysates were reduced in 4 mM dithiothreitol (DTT), alkylated in 8 mM iodoacetamide, and digested sequentially at 37 °C with 1:75 LysC (Wako) and 1:50 Trypsin (Sigma-Aldrich) for 4 and 12 h, respectively. Digested peptides were acidified with 0.1% formic acid (FA) and purified by strong cation exchange STAGE tips, using loading buffer 80% ACN, 0.1% FA and elution buffer 0.5 M ammonium acetate, 20% ACN, 0.1% FA. Eluted peptides were dried by vacuum concentrator and 2 μg equivalent of peptides was analyzed in 3 h reverse phase separation on the UHPLC 1290 system (Agilent) coupled to an Orbitrap Q Exactive Plus mass spectrometer (Thermo Scientific). See Additional file 1: methods for details.

### Immunoblotting

For drug treatment, cells were resuspended in RPMI medium at a density of 1 × 10^6^ cells/ml and incubated for 8 h with the indicated concentrations of BTZ. Cells were lysed in a buffer containing 8 M urea and 50 mM ammonium bicarbonate, supplemented with phosphatase- and protease inhibitor. Protein content was determined using the Bradford assay (Bio-rad) and equal amounts of protein were denatured by boiling in XT Sample buffer (Bio-rad) with 15 mM DTT. Proteins were separated on a 12% % SDS-PAGE gel (Bio-rad) and electroblotted onto PVDF membranes. The antibodies used were 3-phosphoglycerate dehydrogenase (PHGDH, Sigma-Aldrich), phosphoserine-aminotransferase antibody (PSAT1, Abcam), phosphoserine phosphatase antibody (PSPH, Abcam), and Tubulin antibody (Santa Cruz). For patient samples, protein loading was confirmed with a coomassie blue stain of the membrane.

### Multiple myeloma patient samples

CD138-positive cells, isolated from bone marrow of 6 MM patients at diagnosis and during (BTZ) therapy, were available from the Hematology biobank. Patient characteristics are presented in Additional file 1: Table S1. Research was approved by the Medical Ethics Committee of the VU University Medical Center and all patients gave written informed consent.

## Results

### Bortezomib resistance in MM cells is not solely driven by adaptation of the proteasome itself

**Fig. 1:**
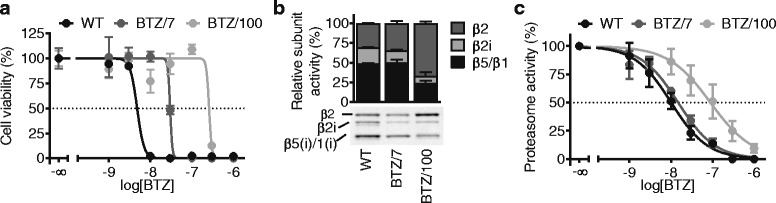
Bortezomib resistance is not solely driven by adaptations of the proteasome. **a** Cell viability of RPMI-8226 wild type (WT) and bortezomib-resistant (BTZ/7 and BTZ/100) cells after a 48-h treatment with increasing concentrations of bortezomib. Results represent % cell viability ± SD compared to non-treated controls of a representative experiment (*n* = 3). **b** In gel fluorescence measurements of a representative experiment showing proteasome activity profiles of RPMI-8226 WT, BTZ/7, and BTZ/100 cells after a 1-h incubation with proteasome activity probe Me_4_BodipyFL-Ahx_3_L_3_VS (lower panel). Quantification of gel images, with subunit activity plotted as of total proteasome activity. Results represent averages of 3 independent experiments (upper panel). **c** Total proteasome activity of RPMI-8226 WT, BTZ/7 and BTZ/100 cells after a 2-h incubation with increasing concentrations of bortezomib, compared to non-treated controls. Results represent quantification of gel images obtained by a 1-h incubation with Me_4_BodipyFL-Ahx_3_L_3_VS. Results represent averages of 3 independent experiments. BTZ = bortezomib

To study BTZ resistance, we compared human BTZ-sensitive RPMI-8226 wild-type (WT) MM cells with two BTZ-resistant cell lines (BTZ/7 and BTZ/100), which grow in the presence of 7 and 100 nM BTZ, respectively [11]. Cell viability assays showed the 48-h IC_50_ values towards BTZ to be 7.3 ± 2.4 nM, 25.3 ± 6.6 nM and 231.9 ± 73 nM in the WT, BTZ/7, and BTZ/100 cell lines, respectively (Fig. 1a), in line with previous reports [11, 33]. Comparable IC_50_ values were found by analyzing the cell growth of these cell lines, and also cell death assays using propidium iodide showed similar trends (Additional file 1: Figure S1a). BTZ-resistant cells displayed cross-resistance towards the proteasome inhibitor carfilzomib (CFZ), but not to the chemotherapeutic drugs methotrexate (folate antagonist) and melphalan (alkylating agent) (Additional file 1: Figure S1b). This suggests that the observed adaptations are related to proteasome inhibitor resistance rather than multidrug resistance, as also previously described for THP-1 cells [10].

Next, we evaluated the proteasome activity in these cells with and without BTZ treatment using the cell-permeable fluorescent proteasome activity probe Me_4_BodipyFL-Ahx_3_L_3_VS. In this assay, the fluorescence intensity directly correlates to the proteasome (subunit) activity, which can be visualized using SDS-PAGE [30]. Without BTZ treatment, both BTZ/7 and BTZ/100 cells showed reduced activity of the β2i subunit, a relative upregulation of the β2 activity and a lower total proteasome activity compared to WT cells (Fig. 1b), consistent with published reports [11, 33, 34]. BTZ treatment decreased total proteasome activity in a dose dependent manner with 50% proteasome inhibition at 107.5 ± 56 nm for the BTZ/100 cells and 10.3 ± 4.1 nM and 15.1 ± 0.9 nM in the WT and BTZ/7 cells, respectively (Fig. 1c, Additional file 1: Figure S1c). Whereas the onset of β2 inhibition was shifted towards higher BTZ concentrations in the BTZ/100 cells, only small differences were observed in the onset of β1/5 inhibition between sensitive and resistant cell lines (Additional file 1: Figure S1c). These results suggest that upregulation of β2 activity is one of the main proteasomal driving forces of BTZ resistance in RPMI-8226 cells. However, in contrast to WT cells, both resistant cell lines still show survival at 50% proteasome inhibition, in line with earlier results [29]. This indicates that in order to survive BTZ treatment, BTZ/7, and BTZ/100 cells not only change the proteasome itself, but also adapt to survive under conditions of continuous proteasome inhibition.

### Bortezomib-resistant cells have an enhanced activity of the pentose phosphate pathway

**Fig. 2:**
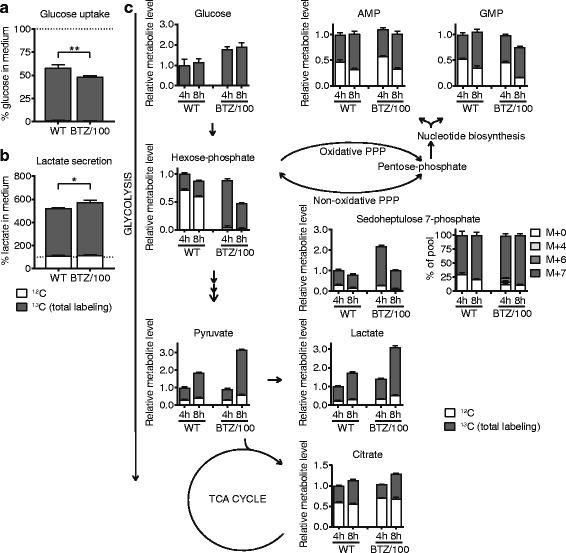
Bortezomib-resistant cells have an enhanced activity of the pentose phosphate pathway. Intra- and extracellular metabolite analysis of RPMI-8226 wild type (WT) and bortezomib resistant (BTZ/100) cells. Cells were suspended in DMEM containing 8 mM [U-^13^C] D-glucose. **a, b** Media samples were collected after 8 h, followed by LC-MS analysis of extracellular glucose (**a**) and lactate (**b**). Results represent % peak area ± SD compared to cell-free media (*n* = 3). Unpaired t-tests were performed (* = *p* < 0.05, ** = *p* < 0.01,). **c** Intracellular metabolites were extracted after 4 and 8 h and analyzed by LC-MS. Data are means ± SD (*n* = 3) of unlabeled (white) and ^13^C- labeled metabolites (gray). AMP = adenosine monophosphate, GMP = guanosine monophosphate, PPP = pentose phosphate pathway, TCA = tricarboxylic acid

Because metabolic alterations are increasingly recognized as a driving force in drug resistance, we next investigated whether such metabolic changes drive adaptation to continuous proteasome inhibition in BTZ-resistant MM cells. BTZ sensitivity has been linked to higher glycolytic activity under hypoxic conditions [28]. However, many metabolic pathways branch off from glycolysis, including the tricarboxylic acid (TCA) cycle, pentose phosphate pathway (PPP) and nucleotide synthesis pathway. Hence, to get a more comprehensive insight into the altered glucose metabolism in BTZ resistant cells, we first mapped the glucose metabolism in both resistant and sensitive cells by performing ^13^C–glucose tracer experiments. To this end, WT and BTZ/100 cells were grown in the presence of 8 mM [U-^13^C]-glucose and the incorporation of ^13^C–carbon from glucose in downstream metabolites was followed over time using mass spectrometry. In addition, changes in extracellular metabolites levels were analyzed. A centrifugation step—which disturbs the cells—is unavoidable to resuspend cells in [U-^13^C]-glucose medium and cells will only reach pseudo-steady state hours after being resuspended. Therefore, early time points (less than 4 h) were excluded from the analysis. As expected, BTZ/100 cells displayed both an increased uptake of glucose from and increased secretion of lactate into the culture medium, indicative of a higher glycolytic activity (Fig. 2a, b). In addition, BTZ/100 cells displayed higher intracellular levels of ^13^C–glucose, as well as higher levels of ^13^C–pyruvate and ^13^C–lactate (Fig. 2c), supporting higher glycolytic activity. No differences in the amount of ^13^C–citrate were observed between cell lines, suggesting no change in glucose flux to the TCA cycle (Fig. 2c). In contrast, sedoheptulose-7-phosphate (S7P), an intermediate of the PPP, was one of the most upregulated metabolites in BTZ/100 cells compared to WT cells. Substantially increased levels of de novo synthesized ^13^C_7_ –labeled S7P (M+7) were present in BTZ/100 cells, indicating a higher PPP activity in resistant cells (Fig. 2c). Moreover, higher amounts of M+4 and M+6 isotopomers of S7P were present in BTZ/100 cells, which are indicative of increased PPP cycling and further support the higher PPP activity in these cells. After 8 h, the S7P levels dropped in both cell lines, suggesting that the cells temporarily raised their PPP activity in response to some type of stress, most likely the centrifugation of the cells. We therefore hypothesize that bortezomib-resistant cells have a higher basal PPP activity as well as a higher capability to increase PPP activity in response to stress [35].

To investigate whether these findings are general features of BTZ-resistant cells, we repeated the MS analysis on BTZ-sensitive AMO-1 multiple myeloma cells and its BTZ- and CFZ-resistant counterparts and included RPMI-8226 cells as a reference. We also included BTZ-resistant ARH-77 cells, which have a different origin (plasmocytoid lymphoma) in these analyses (Additional file 1: Figure S2a-c) [13, 29]. Whereas BTZ-resistant AMO-1 and RPMI-8226 cells all showed higher glucose uptake, lactate secretion and PPP activity compared to sensitive cells, AMO-1 CFZ/90 cells did not show these metabolic changes. Notably, overexpression of the drug efflux transporter ABCB1/P-glycoprotein, for which carfilzomib is a bona fide substrate, is an important underlying mechanism of carfilzomib resistance in CFZ/90 AMO-1 [29, 36]. It is thus likely that these cells do not require metabolic adaptations to oppose proteasome inhibitor induced apoptosis. ARH-77 BTZ-resistant cells on the other hand showed an opposite effect on glucose uptake, lactate secretion and PPP metabolism. Together, these findings show that BTZ-resistant MM cells have higher PPP activity compared to sensitive cells, and suggest that this effect is specific for bortezomib as well as for multiple myeloma.

### Enhanced pentose phosphate pathway activity increases the anti-oxidant capacity of bortezomib-resistant cells

**Fig. 3:**
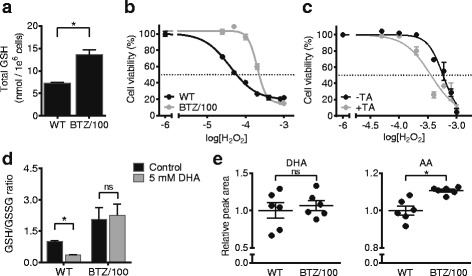
Enhanced pentose phosphate pathway activity increases the anti-oxidant capacity of bortezomib-resistant cells. **a** Total GSH levels of RPMI-8226 wild type (WT) and bortezomib resistant (BTZ/100) cells. Data are means ± SD (*n* = 3) **b** Cell viability of RPMI-8226 WT and BTZ/100 cells after a 16-h treatment with increasing concentrations of H_2_O_2_. Results represent mean cell viability ± SD compared to non-treated controls (*n* = 3). **c** Cell viability of RPMI-8226 BTZ/100 cells after treatment for 24 h with 50 μM trans-androsterone (TA), followed by 24 h with increasing concentrations of H_2_O_2_. Results represent mean cell viability ± SD compared to non-treated control (*n* = 3). **d** GSH/GSSG ratio of RPMI-8226 WT and BTZ/100 cells after 5 min treatment with 5 mM DHA. Data are means ± SD (*n* = 3). **e** Analysis of intracellular levels of DHA (left panel) and AA (right panel) in RPMI-8226 WT and BTZ/100 cells. Cells were treated with 5 mM DHA or vehicle for 5 min and subjected to LC-MS analyses. Data are means ± SD (*n* = 3). Two-way ANOVA tests were performed (ns = not significant, * = *p* < 0.05). AA = ascorbic acid, DHA = dehydroascorbic acid, GSH = glutathione, TA = trans-androsterone

The PPP provides both ribose for purine nucleotide synthesis and NADPH, which cells use for maintaining intracellular redox balance [35]. Since oxidative stress plays a role in the mechanism of action of BTZ, and BTZ/100 cells showed only moderately increased levels of the purines AMP and GMP (Fig. 2c), we hypothesized that BTZ-resistant cells increase their PPP activity predominantly to increase their anti-oxidant defenses and counteract the effect of the drug. Indeed, BTZ-resistant RPMI-8226 cells showed significantly higher concentrations of total glutathione, an important cellular antioxidant (Fig. 3a). Significantly higher levels of intracellular reduced glutathione (GSH) were also found in BTZ-resistant AMO-1 cells as compared to BTZ-sensitive AMO-1 cells (Additional file 1: Figure S2d). RPMI-8226 BTZ/100 cells were also less sensitive to hydrogen peroxide (H_2_O_2_) compared to WT cells (IC_50_ 256 μM vs. 67 μM) (Fig. 3b). When BTZ/100 cells were exposed to H_2_O_2_ after a 24-h pre-incubation with the PPP inhibitor trans-androsterone (TA), the IC_50_ of H_2_O_2_ shifted from 579 μM to 358 μM (Fig. 3c), confirming that resistance to oxidizing agents in these cells is at least partly mediated through their increased PPP activity. Furthermore, we found that the GSH/GSSG ratio was increased in BTZ/100 cells compared to WT cells (Fig. 3d), indicative of an increased anti-oxidant capacity. Finally, the ability of cells to reduce dehydroascorbate (DHA, oxidized vitamin C) to ascorbic acid (AA) can be used as a tool to analyze cellular redox status [37]. DHA is rapidly taken up by the glucose transporters GLUT1 and GLUT3 and subsequently reduced either spontaneously or by GSH- and NADPH-dependent enzymes. As a consequence, DHA reduction is coupled to the ability of cells to regenerate NADPH and GSH [37]. We therefore developed an MS assay to measure the intracellular reduction of DHA to AA (Fig. 3e). Exposure to DHA resulted in similar intracellular levels of DHA in both WT and BTZ/100 cells. In contrast, significantly higher levels of AA were found in BTZ-resistant cells, indicating an increased conversion of DHA to AA. Because reduction of DHA will help maintain a DHA gradient over the cell membrane that favors transport [38], total DHA plus AA levels are also higher in the BTZ-resistant cells. Moreover, whereas DHA exposure decreased GSH/GSSG ratio in WT cells compared to control conditions, this ratio remained stable in BTZ/100 cells (Fig. 3d). These data indicate that the WT cells lack the capacity to regenerate GSH and NADPH, which renders them more sensitive to oxidative stress compared to the resistant cells.

### Bortezomib-resistant cells have a higher activity of the serine synthesis pathway

**Fig. 4:**
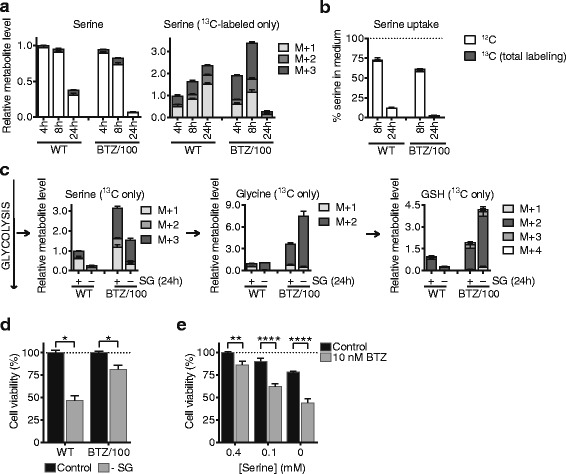
Bortezomib resistant cells have a higher activity of the serine synthesis pathway. **a, b** Intra- and extracellular analysis of serine levels of RPMI-8226 wild type (WT) and bortezomib resistant (BTZ/100) cells. Cells were suspended in DMEM containing 8 mM [U-^13^C] D-glucose. Intracellular metabolites were extracted after 4, 8 and 24 h and analyzed by LC-MS (**a**). Data are means ± SD (*n* = 3) of unlabeled (white) and ^13^C- labeled serine (left panel). Labeled serine was plotted as M+1, M+2 and M+3 isotopomers (right panel). Media samples were collected after 8 and 24 h, followed by LC-MS analysis of extracellular serine (**b**). Results represent % peak area ± SD compared to non-treated media (*n* = 3). **c** Intracellular metabolite analysis of RPMI-8226 WT and BTZ/100 cells in the presence or absence of extracellular serine and glycine (−SG). Cells were grown complete medium in the presence or absence of 0.4 mM serine. After 24 h, media were replaced with matched media containing 20 mM [U-^13^C] D-glucose and intracellular metabolites were extracted after 4 h, followed by LC-MS analysis. Data are means ± SD (*n* = 3) of labeled metabolites. **d** Cell viability of RPMI-8226 WT and BTZ/100 cells after 48 h of serine starvation (−SG). Results represent mean cell viability ± SD compared to non-treated control (*n* = 3). **e** Cell viability of RPMI-8226 WT cells after 48 h in 0.4 mM, 0.1 mM or no serine, including 24 h of 10 nM bortezomib. Results represent mean cell viability ± SD compared to non-treated control (*n* = 3). One-way ANOVA tests were performed (ns = not significant, * = *p* < 0.05, ** = *p* < 0.01, **** = *p* < 0.0001). BTZ = bortezomib, GSH = glutathione

The serine synthesis pathway (SSP), in which serine is synthesized from 3-phosphoglycerate, is another pathway that branches off from glycolysis and that is involved in both NADPH regeneration and GSH production. We therefore questioned whether BTZ-resistant cells also showed increased activity of the SSP. To investigate the basal activity of the SSP in BTZ sensitive and resistant cells, we performed tracer experiments in DMEM containing 8 mM ^13^C–glucose and normal levels of (unlabeled) ^12^C–serine. As the activity of the SSP is controlled primarily by the demand for serine and is only activated when serine is depleted from the extracellular environment [31, 39–43], we expected only low levels of de novo serine synthesis under these conditions. Indeed, both RPMI-8226 WT and BTZ/100 cells contained predominantly ^12^C serine, obtained from the culture medium, and only small amounts of ^13^C–serine (M+1, M+2, and M+3) (Fig. 4a). However, the levels of M+3 serine, which is synthesized de novo from glucose through the SSP [44], were higher in resistant cells compared to wild-type cells, indicative of a higher basal activity of the SSP in these cells. Moreover, BTZ/100 cells displayed a higher uptake of serine from the cell culture medium compared to the WT cells (Fig. 4b). Serine was almost depleted from the culture media of BTZ/100 cells after 24 h, resulting in substantially lower serine levels 24 h after fresh medium was added to the cells (Fig. 4a, b). Although the intracellular data should be interpreted with care, as other nutrients are also depleted at 24 h, these data do indicate that BTZ/100 cells have an increased demand for serine compared to WT cells, which is met by increasing both serine synthesis and serine uptake.

Next, we investigated how BTZ-sensitive and -resistant cells behaved under conditions of serine starvation, when upregulation of the SSP would be a requirement to survive. To this end, cells were grown in the presence or absence of serine and glycine (SG), first for 24 h in the presence of [U-^12^C]-glucose, and subsequently for 4 h using [U-^13^C]-glucose, as described elsewhere [31]. To reduce extra stress on the cells and ensure ample glucose supply throughout the experiment, we supplemented the cell culture medium with 25 mM glucose instead of 8 mM. These higher glucose levels did however not affect serine synthesis, at least under serine-fed conditions (compare Fig. 4a, 4-h time point, to Fig. 4c and Additional file 1: Figure S3a). In both WT and BTZ/100 cells, serine starvation resulted in a decrease in both total serine levels and ^13^C–serine levels (Fig. 4c, Additional file 1: Figure S3a), probably reflecting the rapid conversion of serine to further downstream metabolites [39]. Although total glycine and GSH levels also decreased in both cell lines during serine starvation (Additional file 1: Figure S3a), higher levels of ^13^C–glycine and ^13^C–GSH were found in BTZ/100 cells (Fig. 4c), indicating that the SSP can be activated to a higher extent in resistant cells compared to WT cells. Additionally, BTZ-resistant cells were able to maintain higher levels of GSH during serine starvation (Additional file 1: Figure S3a), supporting our findings that they have higher anti-oxidant capacity.

Given that cells with high SSP activity are able to proliferate in the absence of extracellular serine [44], we next hypothesized that especially WT cells, that have low SSP activity, would be sensitive to serine starvation both in the absence and presence of BTZ. Indeed, serine starvation lowered the viability of WT cells to less than 50%, while BTZ/100 cells remained over 75% viable after 48 h of serine starvation (Fig. 4d), likely because they compensate for the lack of serine through serine synthesis. At the same time, serine starvation significantly increased the amount of cell death in WT cells, but not in BTZ/100 cells (Additional file 1: Figure S3b). Importantly, a 24-h serine starvation prior to BTZ treatment increased the cytotoxic effect of BTZ on WT cells (Fig. 4e). These data not only confirm that the presence of serine is important in the defense mechanism of cells against BTZ, but also suggests that serine starvation could be combined with BTZ to increase the efficacy of treatment in BTZ-sensitive patients.

### Bortezomib resistance correlates to the expression of PHGDH

**Fig. 5:**
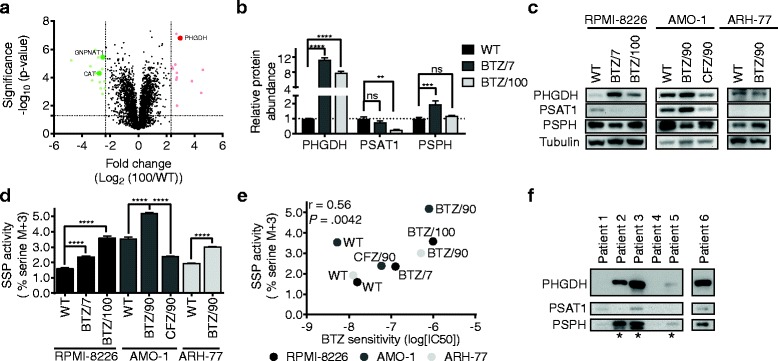
Bortezomib resistance correlates to the expression of PHGDH. **a** Graphical representation of quantitative proteomics data. Proteins are ranked in volcano plot according to their statistically *p*-value (y-axis) and relative abundance ratio between RPMI-8226 wild type (WT) and bortezomib resistant (BTZ/100 cells) (x-axis). Colored spots represent significantly upregulated (red) or downregulated (green) proteins in BTZ/100 cells with at least a 5-fold change. Significantly regulated metabolic enzymes are marked. **b** Quantitative proteomics data of enzymes involved in the serine synthesis pathway. Data represents means ± SD (*n* = 3). **c** Immunoblot of 3-phosphoglycerate dehydrogenase (PHGDH), phosphoserine aminotransferase 1 (PSAT1), phosphoserine phosphatase (PSPH) and Tubulin expression in RPMI-8226, AMO-1 and ARH-77 bortezomib-sensitive and -resistant cells. **d** Fractions of serine M+3 in RPMI-8226, AMO-1 and ARH-77 bortezomib-sensitive and -resistant cells. Data represent % serine M+3 of total serine ± SD (*n* = 3). One-way ANOVA tests were performed (**** = *p* < 0.0001). **e** Correlation between serine M+3 fractions and bortezomib sensitivity in RPMI-8226, AMO-1 and ARH-77 bortezomib-sensitive and -resistant cells. IC_50_s were determined in cell viability assay after 48 h of increasing concentrations of bortezomib. Pearson correlation *r* = 0.56 (*p* = .0042). **f** Immunoblot of PHGDH, PSAT1 and PSPH in isolated CD138+ plasma cells from diagnosed multiple myeloma patients (*n* = 6). Patients with progressive disease/refractory to BTZ-containing therapy are indicated with *. PHGDH is blotted on a separate membrane. BTZ = bortezomib, CFZ = carfilzomib, PHGDH = 3-phosphoglycerate dehydrogenase, PSAT1 = phosphoserine aminotransferase 1, PSPH = phosphoserine phosphatase, SSP = serine synthesis pathway

Having established the importance of both the PPP and SSP in the response of cells to BTZ, we next asked whether these differences were also found on the protein level. Hence, we performed proteomics on WT, BTZ/7 and BTZ/100 cells and quantified >3500 proteins, 395 of which were classified as metabolic enzymes (Fig. 5a, Additional file 1: Figure S4a, Tables S2 and S3). The highest upregulated metabolic enzyme in both BTZ/7 and BTZ/100 cells was 3-phosphoglycerate dehydrogenase (PHGDH; fold change (FC) = 11.27 and 7.86, respectively), the first and rate-limiting enzyme in the SSP, underscoring the importance of the SSP in the response to BTZ. In agreement with our findings, overexpression of PHGDH has previously been associated with an increased glucose flux through the SSP and the ability to proliferate in the absence of extracellular serine [44, 45]. Of the other SSP enzymes, phosphoserine aminotransferase 1 (PSAT1) was downregulated in BTZ/100 cells, while the proteomics data showed no change in the expression of phosphoserine phosphatase (PSPH) in BTZ/100 cells compared to WT cells (Fig. 5b). However, since PHGDH is the rate-limiting enzyme in the SSP, the much higher PHGDH levels in BTZ/100 cells should allow for higher rates of serine synthesis in these cells. An 8-h treatment with BTZ had no effect on SSP enzyme levels in either cell line (Additional file 1: Figure S4b), suggesting that the higher levels of PHGDH in BTZ/100 cells are not resulting from decreased proteasomal degradation of PHGDH but rather from an increase in PHGDH protein synthesis. Amongst the moderately upregulated metabolic enzymes (FC 1.5–5) in BTZ/100 cells were 6-phosphogluconolactonase (PGLS; FC = 1.61) and transketolase (TKT1; FC = 1.53), enzymes involved in NADPH production through PPP. In addition, glutamate-cysteine ligase (GCLC; FC = 1.51) and cystathione γ-lyase (CTH; FC = 2.27), both important enzymes in GSH synthesis, were upregulated. These results are in line with the increased anti-oxidant capacity of resistant cells and further validate the metabolomics results described above.

To validate that higher expression of PHGDH as well as higher SSP activity are general features of BTZ- resistant cells, we performed a western blot for all SSP enzymes on our panel of BTZ- and CFZ-resistant cells (Fig. 5c). In all resistant cell lines, one or more SSP enzymes were upregulated. Both BTZ-resistant RPMI-8226 and AMO-1 cells showed higher expression of PHGDH compared to their sensitive counterparts, AMO-1 BTZ/90 cells showed increased expression of PSAT1, while ARH-77 BTZ/90 cells showed higher expression of PSPH (Fig. 5c). In addition, we profiled the activity of the SSP in these 8 cell lines by measuring the abundance of ^13^C_3_-serine (M+3) after incubation with [U-^13^C]-glucose (Fig. 5d). All BTZ-resistant cells showed a higher M+3 serine fraction compared to their WT counterparts, in line with higher expression of SSP enzymes in BTZ-resistant cells. Moreover, we observed a significant correlation between the serine M+3 fraction and the 48-h IC_50_ towards BTZ in these cell lines (Fig. 5e). In the RPMI-8226 and AMO-1 BTZ-resistant cells, the increase in SSP activity was accompanied by an increased uptake of extracellular serine (Additional file 1: Figure S4c). However, no difference in serine uptake was seen in ARH-77 cells, and no significant correlation was found between serine uptake and the 48-h IC_50_ towards BTZ (Additional file 1: Figure S4d). Together, these data suggest that the ability to perform serine synthesis rather than serine uptake is an important metabolic determinant for BTZ resistance. As expected, CFZ/90 AMO-1 cells did not show an increase in PHGDH expression or serine synthesis (Fig. 5c, d).

To verify the correlation between SSP and BTZ sensitivity, PHGDH expression was examined in CD138+ plasma cells from a small set (*n* = 6) of multiple myeloma patients isolated either at diagnosis, during therapy or after relapse from various therapeutic interventions (Fig. 5f, Additional file 1: Figure S3e, f and Table S1). For one MM patient (#1) at diagnosis, and for one patient (#4) with metastatic disease prior to therapy, PHGDH expression was negligible. Interestingly, three patients with progressive disease or refractory to BTZ therapy (indicated with *) showed markedly increased PHGDH expression, which was accompanied by higher PSPH expression but not PSAT1 expression. Notably, patient #6, that never received BTZ, but relapsed on melphalan, prednisone, lenalidomide therapy also displayed high PHGDH and PSPH expression. These results suggest that (BTZ)-therapy resistance in MM is associated with increased PHDGH expression in CD138+ cells.

## Discussion

Metabolic alterations may play an essential role in the development of cellular resistance against anticancer drugs [24–27]. In the present study, we therefore aimed to identify metabolic mechanisms underlying BTZ resistance in MM. Here, we demonstrate for the first time that BTZ resistance in MM cells is sustained by metabolic rewiring, particularly of serine metabolism. Using tracer-based metabolomics, we show that the SSP has significantly increased activity in BTZ-resistant MM and plasmocytoid lymphoma cell lines. Importantly, we also observed a strong correlation between SSP activity and the ability of cells to withstand increasing BTZ concentrations in all BTZ-resistant cell lines tested.

The SSP is involved in NADPH regeneration and in the production of precursors for nucleotide and glutathione synthesis, and has been shown to be an important pathway to sustain cancer growth and proliferation [42, 43]. PHGDH, the first and rate-limiting enzyme of the SSP, is often overexpressed in cancer and has been linked to tumorigenesis as well as poor diagnosis in different cancers [45–48], identifying PHGDH as an interesting pharmaceutical target [47, 49, 50]. Using proteomics, we show that PHGDH is the highest upregulated metabolic enzyme in BTZ-resistant RPMI-8226 MM cells, and Western blots confirm that also in other BTZ-resistant cell lines one or more SSP enzymes are upregulated. The status of known regulators of the SSP in bortezomib resistance remains to be investigated.

We also demonstrate that PHGDH and PSPH are overexpressed in a small panel of CD138+ cells from BTZ-refractory MM patients compared to BTZ-responsive patients. This raises the possibility that high PHGDH expression, possibly in combination with PSPH expression, could be predictive of BTZ resistance. In line with these observations, we find that serine starvation enhances the efficacy of BTZ in sensitive RPMI-8226 cells, which have not yet upregulated PHGDH. This finding suggests that by removing serine from the diet, which is tolerated by mice and has been shown to reduce growth of some tumors [31, 51], the efficacy of current BTZ treatment might be enhanced. Because patient sample size was limited in the current pilot study, confident conclusions about the role of PHGDH as a predictive biomarker for bortezomib response cannot be drawn at this point. However, the results in patient-derived CD138+ cells underscore our findings in cell lines and warrant further research with a larger, controlled patient cohort to validate the use of PHGDH as a diagnostic tool to determine BTZ sensitivity in MM and other hematologic diseases. Notably, one patient that never received BTZ but was relapsing on melphalan, prednisone and lenalidomide, also displayed high PHGDH expression, raising the possibility that the observed changes in metabolism are also associated with other ROS-inducing agents. BTZ-resistant RPMI-8226 cells indeed show cross-resistance to CFZ, as has also been reported for AMO-1 cells [29], suggesting that the observed metabolic changes confer cross-resistance to other proteasome inhibitors to a certain extent, as expected. However, our data also show that BTZ-resistant RPMI-8226 cells do not display cross-resistance to either methotrexate or melphalan, both of which increase intracellular ROS levels, suggesting that the observed metabolic changes are specific for proteasome inhibitor resistance.

In addition to changes in the SSP, we find that the influx of glucose into the PPP is increased in BTZ-resistant MM cell lines. Proteomics experiments confirm that several proteins involved in both the PPP and in GSH metabolism are upregulated in BTZ resistant cells, in line with previous reports [29]. These metabolic alterations ultimately result in an increased anti-oxidant capacity of BTZ-resistant cells, as evidenced by a lower susceptibility to H_2_O_2_-induced oxidative stress, a higher GSH/GSSG ratio, and an increased ability to convert DHA to AA. Likely, this increased anti-oxidant capacity functions to protect resistant cells from BTZ-induced oxidative stress [6, 52]. Remarkably, increased PPP metabolism was not observed in ARH-77 plasmocytoid lymphoma cells. A recent report shows that BTZ resistance in leukemia was mediated through exocytosis of polyubiquitinated proteins [20], which may eliminate the need for extensive metabolic rewiring to induce resistance. The same holds true for CFZ-resistant cells, in which overexpression of ABCB1/P-glycoprotein has been described as an important mechanism of resistance [29]. It thus seems likely that the ability of cells to employ other mechanisms of resistance regulates the extent of metabolic resistance. Altered serine metabolism, however, occurred in all resistant cell lines tested. Our data therefore strongly support the hypothesis that altered serine metabolism is one of the core mechanisms of resistance, together with changes in proteasome abundance and composition. The clear correlation that we observe between PHGDH expression, SSP activity and BTZ sensitivity encourage further studies to determine whether increased SSP activity is causally related to BTZ resistance and whether PHGDH can be targeted to overcome BTZ resistance.

## Conclusions

Here, we have identified specific metabolic mechanisms underlying bortezomib resistance. In particular, our results imply that bortezomib resistance is associated with high activity of the serine synthesis pathway and overexpression of PHGDH. Interfering with serine metabolism, either by removal of serine from the diet or by PHGDH inhibition, could potentially increase the efficacy of bortezomib treatment in both sensitive and resistant patients. In addition, we propose PHGDH expression as a novel biomarker of bortezomib response.

## Additional file

Additional file 1: Supplemental information. Supplemental Methods; **Figure S1.** Response of RPMI-8226 WT, BTZ/7 and BTZ/100 cells to bortezomib and other chemotherapeutic agent; **Figure S2.** Intra- and extracellular metabolite analysis of BTZ- and CFZ-resistant cell lines; **Figure S3.** Response to serine starvation of RPMI-8226 WT and BTZ/100 cells; **Figure S4.** Bortezomib resistance correlates to the expression of PHGDH; **Table S1.** Characteristics of multiple myeloma patients; **Table S2.** Upregulated metabolic enzymes in RPMI-8226 BTZ/100 and BTZ/7 cells compared to WT; **Table S3.** Downregulated metabolic enzymes in RPMI-8226 BTZ/100 and BTZ/7 cells compared to WT. (PDF 6298 kb)

## Abbreviations

AA: Ascorbic acid
BTZ: Bortezomib
CFZ: Carfilzomib
DHA: Dehydroascorbic acid
FBS: Fetal bovine serum
FC: Fold change
GSH: Glutathione
GSSG: Glutathione disulfide
H2O2: Hydrogen peroxide
MEM: Minimal essential medium
MM: Multiple myeloma
PHGDH: 3-phosphoglycerate dehydrogenase
PPP: Pentose phosphate pathway
PSAT1: Phosphoserine aminotransferase 1
PSPH: Phosphoserine phosphatase
S7P: Sedoheptulose-7-phosphate
SSP: Serine synthesis pathway
TA: Trans-androsterone
TCA: Tricarboxylic acid
WT: Wild type
