# Supplementary material for: Bortezomib resistance in multiple myeloma is associated with increased serine synthesis
Source: Cancer Metab. 2017 Aug 29;5:7. doi: 10.1186/s40170-017-0169-9 (PMC5575874; doi:10.1186/s40170-017-0169-9)
Supplement: Additional file 1: — Supplemental information. Supplemental Methods; Figure S1. Response of RPMI-8226 WT, BTZ/7 and BTZ/100 cells to bortezomib and other chemotherapeutic agent; Figure S2. Intra- and extracellular metabolite analysis of BTZ- and CFZ-resistant cell lines; Figure S3. Response to serine starvation of RPMI-8226 WT and BTZ/100 cells; Figure S4. Bortezomib resistance correlates to the expression of PHGDH; Table S1. Characteristics of multiple myeloma patients; Table S2. Upregulated metabolic enzymes in RPMI-8226 BTZ/100 and BTZ/7 cells compared to WT; Table S3. Downregulated metabolic enzymes in RPMI-8226 BTZ/100 and BTZ/7 cells compared to WT. (PDF 6298 kb) [file 40170_2017_169_MOESM1_ESM.pdf]

Supplemental Information

Bortezomib resistance in multiple myeloma is associated with increased serine synthesis

Esther A. Zaal, Wei Wu, Gerrit Jansen, Sonja Zweegman, Jacqueline Cloos

Celia R. Berkers

Supplemental Methods

Supplemental Figures

Supplemental Methods; Supplemental figure S1: Response of RPMI-8226 WT, BTZ/7 and BTZ/100 cells to bortezomib and other chemotherapeutic agent

Supplemental figure S2: Intra- and extracellular metabolite analysis of BTZ- and CFZ-resistant cell lines

Supplemental figure S3: Response to serine starvation of RPMI-8226 WT and BTZ/100 cells

Supplemental figure S4: Bortezomib resistance correlates to the expression of PHGDH

Supplemental Tables

Supplemental Table S1: Upregulated metabolic enzymes in RPMI-8226 BTZ/100 and BTZ/7 cells compared to WT

Supplemental Table S2: Downregulated metabolic enzymes in RPMI-8226 BTZ/100 and BTZ/7 cells compared to WT

Supplemental Table S3: Characteristics of multiple myeloma patients.

Supplemental methods

Liquid chromatrography – Mass Spectrometry based Metabolomics

Metabolites were extracted by adding 100 – 200µl ice-cold MS lysis buffer (methanol/acetonitrile/uLCMS H2O (2:2:1)) to the cell pellets. To measure extracellular metabolites, metabolites were extracted by diluting 10µL medium in 1mL MS lysis buffer. Samples were shaken for 10 minutes at 4°C, centrifuged at 14.000g for 15 minutes at 4°C and supernatants were collected for LC-MS analysis. LC-MS analysis was performed on an Exactive mass spectrometer (Thermo Scientific) coupled to a Dionex Ultimate 3000 autosampler and pump (Thermo Scientific). The MS operated in polarity-switching mode with spray voltages of 4.5kV and -3.5kV. Metabolites were separated on a Sequant ZIC-pHILIC column (2.1 x 150mm, 5µm, Merck) with guard column (2.1 x 20mm, 5µm, Merck) using a linear gradient of acetonitrile and a buffer containing 20mM $(NH_4)_2CO_3$, 0.1% NH_4OH in ULC/MS grade water. Flow rate was set at 150 µL/min. Metabolites were identified based on exact mass within 5 ppm and further validated by concordance with retention times of standards. Metabolites were quantified using LCquan software (Thermo Scientific). Peak intensities were normalized based on median peak intensity.

RP-nanoLC-MS/MS

Proteomics data were acquired using an UHPLC 1290 system (Agilent) coupled to an Orbitrap Q Exactive Plus mass spectrometer (Thermo Scientific). Peptides were first trapped on a 2 cm x 100 µm Reprosil C18 pre-column (3 µm) and then separated on a 50 cm x 75 µm Poroshell EC-C18 analytical column (2.7 µm). Trapping was performed for 10 min in 0.1M acetic acid (Solvent A) and elution with 80% ACN in 0.1M acetic acid (Solvent B) in gradients as follows: 10-36% solvent B in 155 min, 36-100% in 3min and finally 100% for 1min. Flow was passively split to 300 nl/min. MS data were obtained in data-dependent acquisition mode. Full scans were acquired in the m/z range of 375-1600 at the resolution of 35,000 (m/z 400) with AGC target 3E6.Top 15 most intense precursor ions were selected for HCD fragmentation performed at NCE 25% after accumulation to target value of 5E4. MS/MS acquisition was performed at a resolution of 17,500.

Database search

Raw files were processed using MaxQuant version 1.5.3.30 and searched against the human Swissprot database (version Jan 2016) using Andromeda. Cysteine carbamidomethylation was set to fixed modification, while variable modifications of methionine oxidation and protein N-terminal acetylation, as well as up to 2 missed cleavages were allowed. False discovery rate (FDR) was restricted to 1% in both protein and peptide identification. Label-free quantification (LFQ) was performed with “match between runs” enabled.

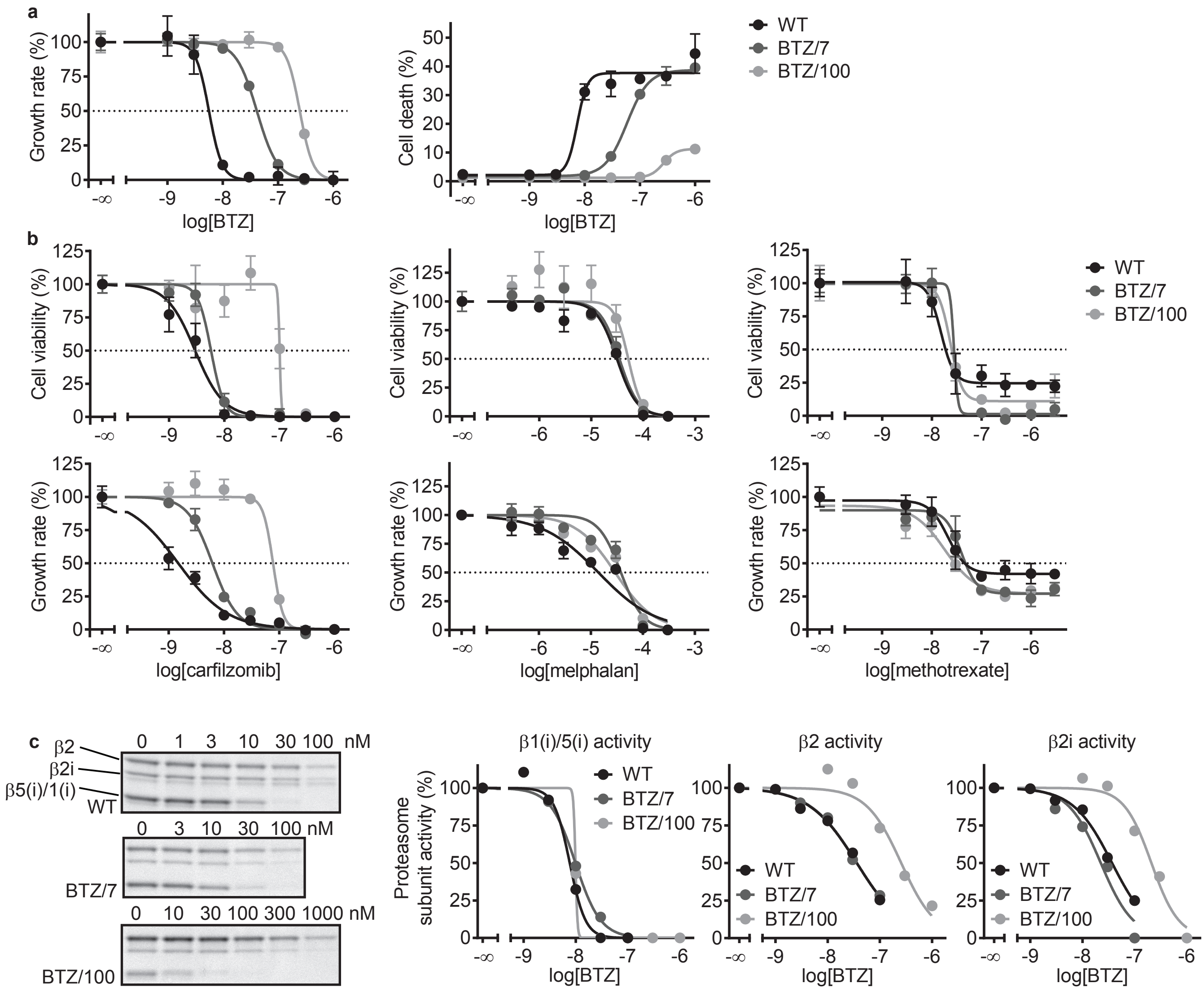

Supplemental figure S1: Response of RPMI-8226 WT, BTZ/7 and BTZ/100 cells to bortezomib and other chemotherapeutic agents

a Growth rate and cell death of RPMI-8226 wild type (WT) and bortezomib=resistant (BTZ/100) cells after a 48-hour treatment with increasing concentrations of bortezomib (BTZ). Results represent % growth rate (left panel) or % cell death (right panel) ± SD compared to non-treated controls (n=3). **b** Cell viability and growth rate of RPMI-8226 wild type (WT) and bortezomib-resistant (BTZ/100) cells after a 48-hour treatment with increasing concentrations of carfilzomib, melphalan and methotrexate. Results represent % cell viability (upper panel) or % growth rate (lower panel) ± SD compared to non-treated controls (n=3). **c** In gel fluorescence measurements showing representative proteasome activity profiles of RPMI-8226 WT, BTZ/7 and BTZ/100 cells after 2 hour of bortezomib treatment in increasing concentrations, including 1 hour treatment with proteasome activity probe $Me_4BodipyFLAhx_3L_3VS$ (left panel). Quantification of gel images, with results plotted as fractions of subunit activity compared to non-treated controls (right panel). BTZ = bortezomib

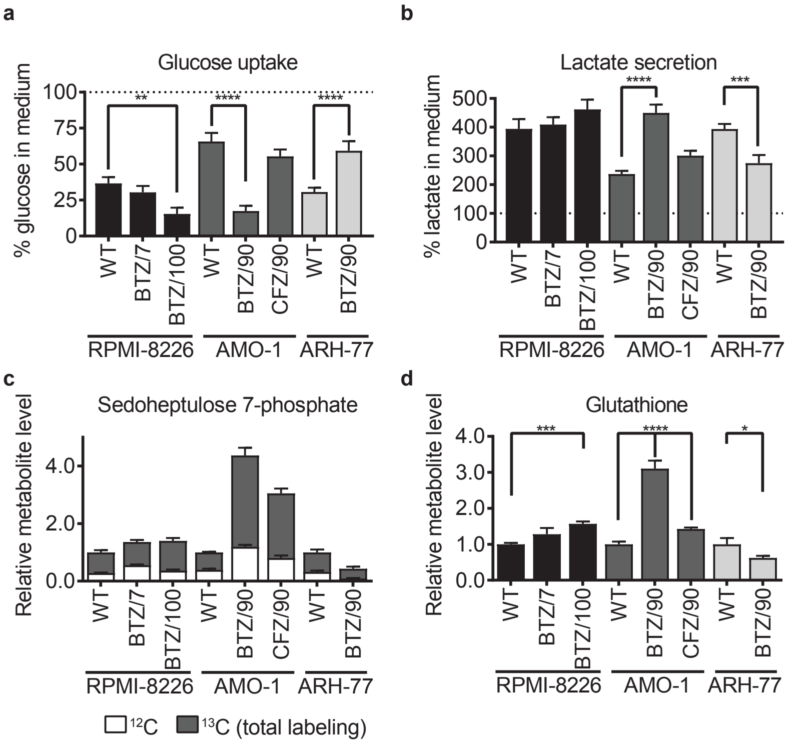

Supplemental figure S2: Intra- and extracellular metabolite analysis of BTZ- and CFZ-resistant cell lines

a,b Extracellular metabolite analysis of RPMI-8226, AMO-1 and ARH-77 wild type (WT) and bortezomib- and carfilzomib-resistant cells. Cells were suspended in MEM with 1mM L-glutamine, 0.2mM L-serine and 0.2mM L-glycine. Media samples were collected after 8 hours, followed by LC-MS analysis of extracellular glucose (**a**) and lactate (**b**). Results represent % peak area ± SD compared to cell-free media (n=3). **c,d** Intracellular metabolite analysis of RPMI-8226, AMO-1 and ARH-77 wild type (WT) and bortezomib- and carfilzomib-resistant cells. Cells were suspended in DMEM containing 8mM [U-^{13}C] D-glucose. Intracellular metabolites were extracted after 8 hours and analyzed by LC-MS. Data are means ± SD (n=3) of unlabelled (white) and ^{13}C- labelled metabolites (grey) for sedoheptulose 7-phosphate (**c**) and total glutathione (**d**). One-way ANOVA tests were performed (* = p<0.05, ** = p<0.01, *** = p<0.001, **** = p<0.0001).

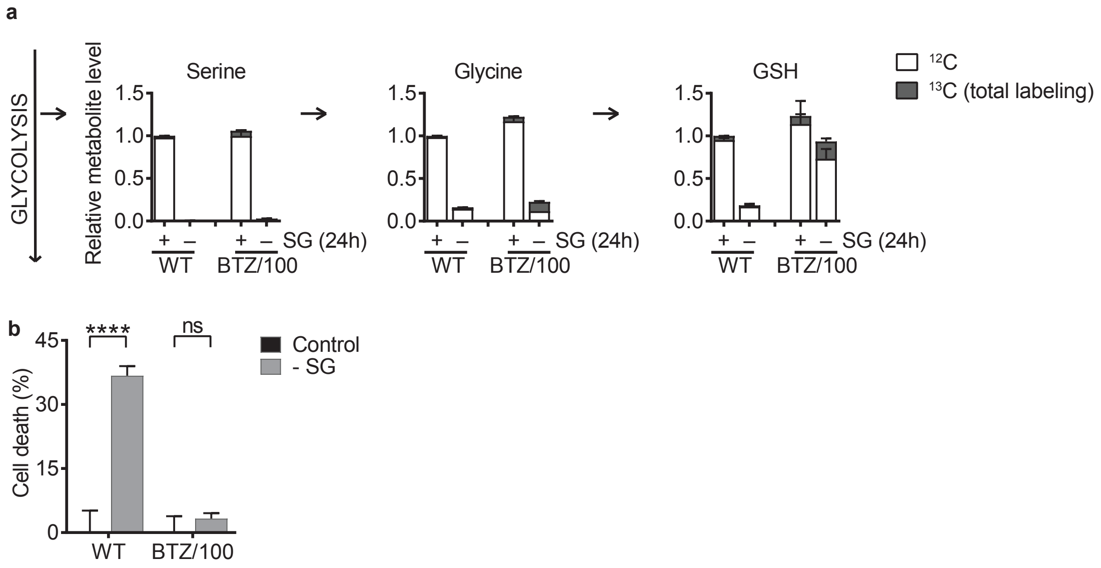

Supplemental figure S3: Response to serine starvation of RPMI-8226 WT and BTZ/100 cells

a Intracellular metabolite analysis of RPMI-8226 WT and BTZ/100 cells in the presence or absence of extracellular serine (-SG). Cells were grown complete medium in the presence or absence of 0.4mM serine. After 24 hours, medium were replaced with matched media containing 20 mM [U-^{13}C]D-glucose. Cells were harvested after 4 hours and subjected to LC-MS analysis. Data are means ± SD (n=3) of unlabelled (white) and labelled metabolites (grey). **b** Cell death assay of RPMI-8226 WT and BTZ/100 cells in the presence or absence of extracellular serine (-SG). Data are means ± SD of % death cells after 24 hours. Two-way ANOVA tests were performed (**** = p<0.0001).BTZ = bortezomib. GSH = glutathione.

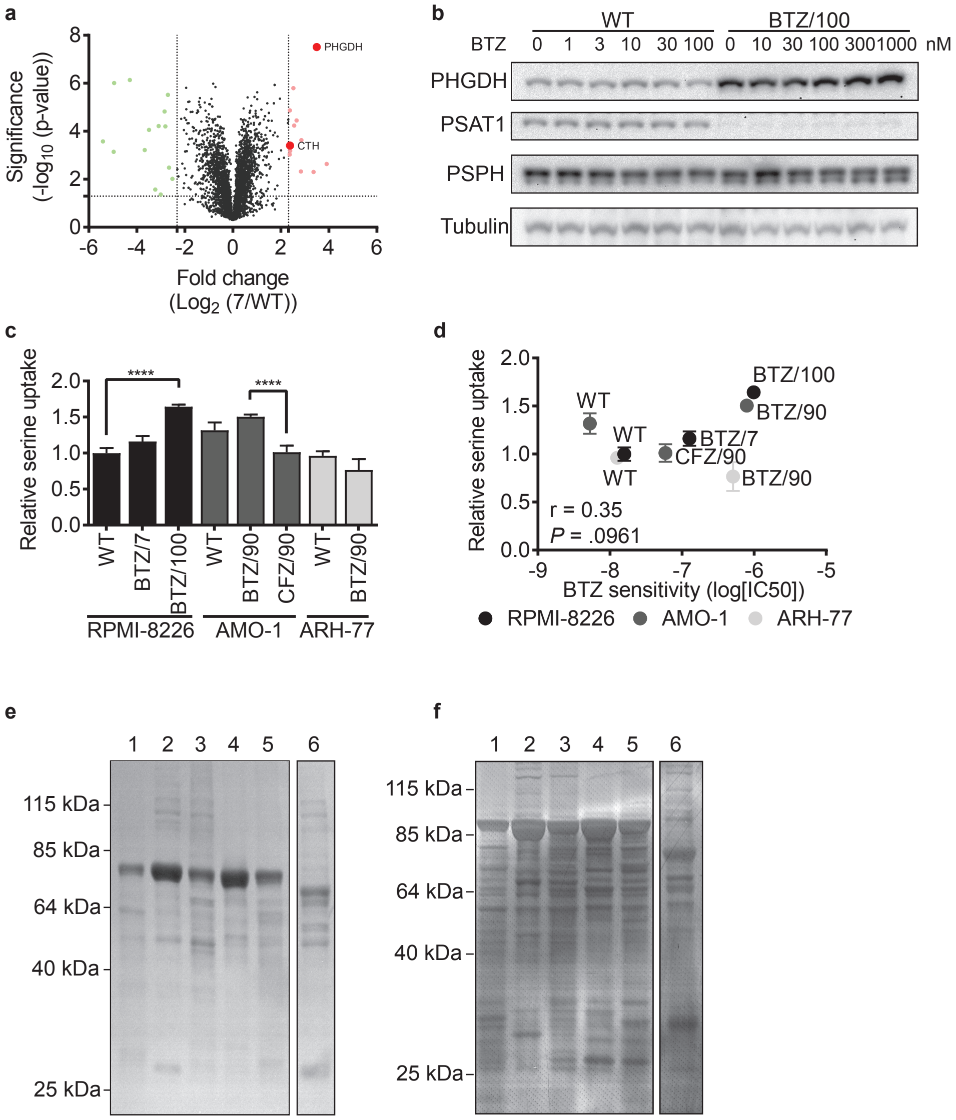

Supplemental figure S4: Bortezomib resistance correlates to the expression of PHGDH

a Graphical representation of quantitative proteomics data. Proteins are ranked in volcano plot according to their statistically p-value (y-axis) and relative abundance ratio between RPMI-8226 WT and BTZ/7 cells (x-axis). Coloured spots represent significantly upregulated (red) or downregulated (green) proteins in BTZ/7 cells with at least a 5-fold change. Significantly regulated metabolic enzymes are marked. **b** Immunoblot of 3-phosphoglycerate dehydrogenase (PHGDH), phosphoserine aminotransferase 1 (PSAT1), phosphoserine phosphatase (PSPH) and Tubulin expression in RPMI-8226 WT and BTZ/100 cells after 8 hour incubation with increasing concentrations of bortezomib. **c** Extracellular metabolite analysis of RPMI-8226, AMO-1 and ARH-77 wild type (WT) and bortezomib- and carfilzomib-resistant cells. Cells were suspended in MEM with 1mM L-glutamine, 0.2mM L-serine and 0.2mM L-glycine. Media samples were collected after 8 hours, followed by LC-MS analysis of extracellular serine. Results represent % peak area ± SD compared to cell-free media (n=3). One-way ANOVA tests were performed (**** = p<0.0001). **d** Correlation between serine uptake and IC50 of bortezomib in RPMI-8226, AMO-1 and ARH-77 bortezomib-sensitive and -resistant sublines. Pearson correlation: r = 0.35 (p=.0961). **e,f** Coomassie stained membrane for loading control of PHGDH (**e**) and PSAT1 and PSPH (**f**) with isolated CD138+ plasma cells from diagnosed multiple myeloma patients (n=6). BTZ = bortezomib, CFZ = carfilzomib, PHGDH = 3-phosphoglycerate dehydrogenase, PSAT1 = phosphoserine aminotransferase 1, PSPH = phosphoserine phosphatase.

Supplemental Table S1: Upregulated metabolic enzymes in RPMI-8226 BTZ/100 and BTZ/7 cells compared to WT

Protein ID	Gene name	Protein name	BTZ/100 : WT		BTZ/7 : WT	
			FC	p-value	FC	p-value
O43175	PHGDH	D-3-phosphoglycerate dehydrogenase	7.86	1.57E-07 *	11.27	3.08E-08 *
Q5T6L4	ASS	Argininosuccinate synthase	3.29	4.57E-06 *	1.61	6.78E-04 *
O60701	UGDH	UDP-glucose 6-dehydrogenase	2.92	1.92E-05 *	2.54	2.34E-05 *
X5D767	ADA	Adenosine deaminase	2.66	1.37E-04 *	2.80	3.60E-05 *
Q16222	UAP1	UDP-N-acetylhexosamine pyrophosphorylase	2.56	4.35E-05 *	3.36	1.17E-06 *
F5H5V4	PSMD9	26S proteasome non-ATPase regulatory subunit 9	2.41	8.44E-05 *	1.16	1.31E-02 *
P16152	CBR1	Carbonyl reductase [NADPH] 1	2.30	1.12E-04 *	1.40	7.60E-02 *
Q6LET3	HPRT1	Hypoxanthine-guanine phosphoribosyltransferase	2.28	7.40E-05 *	1.58	9.22E-03 *
P32929	CTH	Cystathionine gamma-lyase	2.27	1.41E-03 *	5.22	3.94E-04 *
D6RFF8	GNPDA1	Glucosamine-6-phosphate isomerase	2.15	2.87E-03 *	1.40	4.93E-03 *
B3KN05	ACSL3	Long-chain-fatty-acid--CoA ligase 3	2.13	7.59E-04 *	2.70	3.16E-04 *
Q8NFW8	CMAS	N-acylneuraminate cytidylyltransferase	2.06	5.09E-04 *	1.64	7.62E-04 *
E9PNU1	NME7	Nucleoside diphosphate kinase 7	1.97	2.51E-03 *	1.33	4.31E-02 *
A0A024R275	RFK	Riboflavin kinase	1.95	1.75E-03 *	1.10	1.21E-01
H0YMB3	GMPR2	GMP reductase	1.92	1.91E-05 *	2.03	1.25E-04 *
A0A024R912	UCK2	Uridine kinase	1.88	1.72E-03 *	1.60	1.65E-03 *
Q86TP1	PRUNE	Protein prune homolog	1.87	1.53E-04 *	2.18	5.81E-03 *
A8K8N7	PFAS	Phosphoribosylformylglycinamidine synthase	1.85	5.26E-04 *	1.90	2.05E-04 *
Q5M7Z5	GRHPR	Glyoxylate reductase/hydroxypyruvate reductase	1.82	2.07E-03 *	1.25	1.56E-01
P23526	AHCY	Adenosylhomocysteinase	1.81	8.66E-05 *	2.83	1.74E-05 *
B2ZZ90	ACACA	Acetyl-CoA carboxylase 1	1.76	2.49E-05 *	1.48	2.67E-03 *
Q53HM1	UCKL1	Uridine kinase	1.74	5.86E-03 *	1.25	5.64E-02
Q9NZL9	MAT2B	Methionine adenosyltransferase 2 subunit beta	1.74	6.97E-05 *	1.27	5.14E-02
B4DM26	LCLAT1	Lysocardiolipin acyltransferase 1	1.74	3.53E-03 *	1.48	1.00E-02 *
P28070	PSMB4	Proteasome subunit beta type-4	1.73	2.47E-03 *	0.78	2.41E-01
B7Z1Y2	ALDOC	Fructose-bisphosphate aldolase	1.72	1.86E-04 *	3.28	6.84E-06 *
P28074	PSMB5	Proteasome subunit beta type-5	1.71	3.93E-04 *	1.28	3.02E-02 *
Q504W7	OCRL	Inositol polyphosphate 5-phosphatase OCRL-1	1.70	3.08E-03 *	1.33	2.59E-02 *
B2R9X3	GMDS	GDP-mannose 4,6 dehydratase	1.68	3.27E-04 *	2.15	5.15E-05 *
P08243	ASNS	Asparagine synthetase	1.67	1.14E-04 *	1.52	6.16E-03 *
X5D8S6	ADSL	Adenylosuccinate lyase	1.66	1.09E-03 *	1.01	4.86E-01
P52788	SMS	Spermine synthase	1.66	2.50E-04 *	1.25	1.92E-02 *
A0A024QZW7	NUP153	Nuclear pore complex protein Nup153	1.63	2.42E-05 *	1.63	1.66E-05 *
P15531	NME1	Nucleoside diphosphate kinase A	1.62	1.16E-02 *	1.57	3.01E-02 *
A8K6Y2	PPP2R5D	Phosphatase 2A, regulatory subunit B56 delta isoform	1.62	1.35E-03 *	1.90	1.37E-04 *
O95336	PGLS	6-phosphogluconolactonase	1.61	7.07E-03 *	1.26	6.40E-02 *
Q96HT3	POLR1C	DNA-directed RNA polymerases I and III subunit RPAC1	1.60	1.21E-03 *	1.37	2.01E-02 *
C9JFR7	CYCS	Cytochrome c	1.59	5.91E-02	2.66	6.90E-03
A0A024R652	MTHFD1	Methylenetetrahydrofolate dehydrogenase	1.58	2.07E-04 *	1.49	1.23E-04 *
P25787	PSMA2	Proteasome subunit alpha type	1.58	3.26E-03 *	1.15	7.93E-02
Q53HS0	QARS	Glutaminyl-tRNA synthetase variant	1.56	1.42E-04 *	1.02	3.90E-01
V9HWD9	TKT1	Transketolase	1.53	8.06E-06 *	0.74	1.95E-03 *
P25325	MPST	3-mercaptopyruvate sulfurtransferase	1.53	4.98E-02 *	1.26	2.01E-01
P12268	IMPDH2	Inosine-5-monophosphate dehydrogenase 2	1.52	5.95E-04 *	0.82	5.20E-04 *
E9PF10	NUP155	Nuclear pore complex protein Nup155	1.51	1.47E-04 *	1.56	1.49E-04 *
Q14TF0	GCLC	Glutamate-cysteine ligase	1.51	7.48E-04 *	1.21	6.59E-02 *

Metabolic enzymes that are upregulated in RPMI-8226 BTZ/100 cells compared to WT cells with fold change (FC) > 1.5

* = p<0.05 based on Student's T-Test

Supplemental Table S2: Downregulated metabolic enzymes in RPMI-8226 BTZ/100 and BTZ/7 cells compared to WT

Protein ID	Gene name	Protein name	BTZ/100 : WT		BTZ/7 : WT	
			FC	p-value	FC	p-value
P04040	CAT	Catalase	0.14	4.98E-05 *	0.65	2.90E-02 *
G3V4W4	A0A024R5K6	Glucosamine 6-phosphate N-acetyltransferase	0.17	3.56E-06 *	0.29	2.11E-04 *
A0A024R5K6	NDUFC2	NADH dehydrogenase [ubiquinone] 1 subunit C2	0.23	5.67E-02	0.54	3.62E-01
J3KS22	DCXR	L-xylulose reductase	0.25	2.32E-04 *	0.25	6.93E-04 *
Q6FGU2	DTYMK	Thymidylate kinase	0.25	1.01E-04 *	0.43	8.87E-05 *
A0A087WXI1	HIBCH	3-hydroxyisobutyryl-CoA hydrolase	0.27	3.06E-05 *	0.40	3.50E-04 *
A0A024R222	PSAT1	Phosphoserine aminotransferase	0.27	6.72E-05 *	0.77	1.75E-02 *
Q9P2X2	SLC1A4	Amino acid transporter	0.27	1.40E-04 *	0.38	4.17E-03 *
B4DEA8	ACADVL	Very long-chain specific acyl-CoA dehydrogenase	0.28	6.96E-04 *	0.21	7.66E-03 *
P36871	PGM1	Phosphoglucomutase-1	0.33	8.47E-05 *	0.35	9.04E-05 *
A0A024QZ78	AGMAT	Agmatinase	0.33	2.67E-02 *	0.36	1.62E-02 *
A0A024RBX9	PDHA1	Pyruvate dehydrogenase E1 component subunit alpha	0.34	5.71E-04 *	0.50	2.72E-03 *
Q53GL5	IDH2	Isocitrate dehydrogenase [NADP]	0.34	1.27E-03 *	0.33	3.21E-04 *
Q71UA6	SLC1A5	Amino acid transporter	0.34	1.63E-01	1.11	2.64E-01
E9PN17	ATP5L	ATP synthase subunit g	0.37	5.76E-03 *	0.71	6.93E-02
A0A024QYX0	EBP	3-beta-hydroxysteroid-Delta(8),Delta(7)-isomerase	0.37	3.30E-04 *	0.48	9.19E-04 *
Q5T0S4	PPT1	Palmitoyl-protein thioesterase 1	0.37	1.93E-04 *	0.63	9.87E-03 *
Q53X12	ATP6V0A1	V-type proton ATPase subunit a	0.40	5.84E-02	0.67	9.44E-03 *
Q8TCJ2	STT3B	Dolichyl-diphosphooligosaccharide-<br>-protein glycosyltransferase subunit STT3B	0.40	1.50E-03 *	0.67	4.23E-02 *
B4DJ81	NDUFS1	NADH-ubiquinone oxidoreductase 75 kDa subunit	0.40	8.00E-03 *	0.67	1.03E-02 *
B3KM58	DKFZp686O15	Putative uncharacterized protein DKFZp686O15119	0.41	9.83E-04 *	0.38	3.97E-04 *
P11177	PDHB	Pyruvate dehydrogenase E1 component subunit beta	0.42	1.50E-03 *	0.38	5.60E-04 *
B2R7W0	MTHFD2	methylenetetrahydrofolate dehydrogenase	0.42	2.35E-03 *	0.62	3.33E-03 *
Q15067	ACOX1	Peroxisomal acyl-coenzyme A oxidase 1	0.42	5.34E-04 *	0.43	6.81E-03 *
A0A024R6G6	NDUFB1	NADH dehydrogenase [ubiquinone] 1 beta subcomplex	0.42	9.24E-02	0.70	7.18E-02
B4E072	ACAA1	3-ketoacyl-CoA thiolase	0.43	1.46E-04 *	0.60	5.84E-03 *
A0A087WTV6	PYCR2	Pyrroline-5-carboxylate reductase 2	0.43	1.62E-03 *	0.53	2.09E-03 *
A0A024QZ30	SDHA	Succinate dehydrogenase [ubiquinone]<br>flavoprotein subunit	0.43	2.46E-05 *	0.70	2.96E-03 *
P22695	UQCRC2	Cytochrome b-c1 complex subunit 2	0.43	2.76E-03 *	0.60	2.66E-02 *
A0A0C4DGS1	DDOST	Dolichyl-diphosphooligosaccharide-<br>-protein glycosyltransferase 48 kDa subunit	0.44	1.81E-04 *	0.77	5.46E-02
P32321	DCTD	Deoxycytidylate deaminase	0.44	2.50E-02 *	0.65	5.71E-03 *
B4DZ08	ACO2	Aconitate hydratase	0.44	4.81E-05 *	0.56	9.45E-04 *
Q13057	COASY	Bifunctional coenzyme A synthase	0.45	2.84E-03 *	0.41	4.68E-04 *
Q5QNZ2	ATP5F1	ATP synthase F(0) complex subunit B1	0.45	1.34E-04 *	0.59	1.11E-03 *
Q2TB59	NNT	NAD(P) transhydrogenase	0.45	7.85E-04 *	0.43	8.22E-03 *
P31930	UQCRC1	Cytochrome b-c1 complex subunit 1	0.46	7.51E-04 *	0.64	1.17E-02 *
P21912	SDHB	Succinate dehydrogenase [ubiquinone]<br>iron-sulfur subunit	0.46	2.57E-03 *	0.73	1.40E-01
Q0QEN7	ATP5B	ATP synthase subunit beta	0.47	6.82E-04 *	0.51	2.83E-03 *
B3KUZ8	GOT2	Aspartate aminotransferase	0.47	7.87E-04 *	0.52	2.83E-03 *
P24752	ACAT1	Acetyl-CoA acetyltransferase	0.47	3.26E-04 *	0.68	6.34E-03 *
A0A024R7A8	AKR1B1	Aldose reductase	0.47	1.69E-04 *	0.75	1.09E-04 *
A8K168	ME1	Malic enzyme	0.48	6.98E-02	0.97	4.81E-01
A0A024R3W5	SLC39A10	Zinc transporter ZIP10	0.48	6.04E-02	0.58	5.93E-02
B4DRH6	HADHA	Long-chain enoyl-CoA hydratase	0.48	3.32E-07 *	0.64	4.16E-04 *
P53602	MVD	Diphosphomevalonate decarboxylase	0.49	4.35E-03 *	0.61	1.42E-02 *
B4DY96	HADHB	3-ketoacyl-CoA thiolase	0.49	4.49E-04 *	0.50	3.64E-03 *
Q53GR7	SLC25A13	Calcium-binding mitochondrial carrier protein Aralar2	0.49	3.91E-02 *	0.77	2.94E-01
B4E0N9	GLUD1	Glutamate dehydrogenase	0.49	5.53E-04 *	0.49	2.03E-03 *
Q53FM7	NDUFS3	NADH dehydrogenase [ubiquinone] iron-sulfur protein 3	0.50	3.71E-03 *	0.91	2.50E-01
D3DVH3	INPP4A	Type I inositol 3,4-bisphosphate 4-phosphatase	0.50	2.01E-03 *	0.68	1.03E-02 *
A0A087WVM4	MTHFD1L	Monofunctional C1-tetrahydrofolate synthase	0.50	2.19E-03 *	0.87	2.11E-01
Q53SW4	NDUFA10	NADH dehydrogenase [ubiquinone] 1 alpha subcomplex sub	0.50	2.94E-03 *	0.59	6.24E-03 *
H7C3G9	NAGK	N-acetyl-D-glucosamine kinase	0.50	3.20E-05 *	0.46	4.39E-04 *
Q6IB54	ATP5J	ATP synthase-coupling factor 6	0.51	3.06E-04 *	0.61	4.60E-03 *
P13073	COX4I1	Cytochrome c oxidase subunit 4 isoform 1	0.51	1.66E-03 *	0.64	6.51E-03 *
A4D1K0	ATP6V1F	V-type proton ATPase subunit F	0.52	1.06E-02 *	0.64	1.46E-03 *
Q5QP19	NFS1	Cysteine desulfurase	0.52	6.18E-04 *	0.66	2.54E-03 *
C9JJU1	SLC38A5	Sodium-coupled neutral amino acid transporter 5	0.52	5.86E-02	0.47	1.53E-01
Q96HA1	POM121	Nuclear envelope pore membrane protein POM 121	0.52	2.77E-04 *	0.68	1.35E-03 *
B2RE46	RPN2	Dolichyl-diphosphooligosaccharide-<br>-protein glycosyltransferase subunit 2	0.52	7.94E-04 *	0.86	4.72E-02 *
B2R659	HSD17B4	Peroxisomal multifunctional enzyme type 2	0.53	1.04E-03 *	0.74	7.46E-03 *
E9PC15	AGK	Acylglycerol kinase	0.53	8.21E-05 *	0.66	5.82E-03 *
P47985	UQCRFS1	;Cytochrome b-c1 complex subunit 11	0.54	1.78E-02 *	0.55	6.43E-02
B7Z9J8	IDH3A	Isocitrate dehydrogenase [NAD] subunit	0.54	1.12E-01	1.65	2.12E-01
O75947	ATP5H	ATP synthase subunit d	0.54	1.49E-03 *	0.67	6.89E-02

B4DFL1	DLD	Dihydrolipoyl dehydrogenase	0.55	1.94E-02 *	0.54	2.65E-03 *
Q0QF37	MDH2	Malate dehydrogenase	0.56	6.85E-04 *	0.59	4.21E-03 *
K7EPM1	ENO3	Enolase	0.56	9.11E-03 *	0.60	4.53E-03 *
E9PDF2	OGDH	2-oxoglutarate dehydrogenase	0.56	1.29E-04 *	0.62	3.95E-03 *
E7D7X9	PYCR1	Pyrroline-5-carboxylate reductase	0.57	2.23E-04 *	0.55	9.36E-06 *
Q6FHM4	COX5B	Cytochrome c oxidase subunit 5B	0.57	1.05E-02 *	0.65	2.35E-02 *
Q9GZS1	POLR1E	DNA-directed RNA polymerase I subunit RPA49	0.58	9.82E-03 *	1.00	5.00E-01
F5GZS6	SLC3A2	4F2 cell-surface antigen heavy chain	0.59	1.61E-03 *	0.75	5.05E-03 *
Q8N4P3	HDDC3	Guanosine-3,5-bis(diphosphate) 3-pyrophosphohydrolase M	0.59	5.10E-03 *	0.97	3.51E-01
O00116	AGPS	Alkyldihydroxyacetonephosphate synthase	0.59	1.94E-03 *	0.83	8.40E-02
B3KNL8	ALG2	Alpha-1,3/1,6-mannosyltransferase ALG2	0.59	4.21E-03 *	0.59	7.66E-03 *
B2R761	SCP2	Non-specific lipid-transfer protein	0.59	9.38E-03 *	0.67	1.83E-02 *
E9PF18	HADH	Hydroxyacyl-coenzyme A dehydrogenase	0.59	1.76E-03 *	0.55	9.66E-04 *
Q99714	HSD17B10	3-hydroxyacyl-CoA dehydrogenase type-2	0.59	1.43E-03 *	0.64	5.16E-03 *
B4DL14	ATP5C1	ATP synthase subunit gamma	0.59	3.99E-03 *	0.64	2.87E-02 *
Q9NRN7	AASDHPPT	L-aminoadipate-semialdehyde dehydrogenase-phosphopantetheinyl transferase	0.60	4.63E-04 *	1.03	4.39E-01
A0A075X871	COX3	Cytochrome c oxidase subunit 3	0.60	2.40E-02 *	0.91	2.60E-01
Q9NR45	NANS	Sialic acid synthase	0.60	1.16E-03 *	0.60	5.31E-03 *
A0A087WUV8	BSG	Basigin	0.61	9.33E-04 *	0.74	1.85E-02 *
A0A087WZN1	IDH3B	Isocitrate dehydrogenase [NAD] subunit	0.61	1.76E-04 *	0.98	3.98E-01
E9PPM8	DERA	Deoxyribose-phosphate aldolase	0.61	6.77E-04 *	0.69	3.45E-03 *
Q53H22	PPAT	Amidophosphoribosyltransferase	0.62	1.89E-04 *	0.58	3.24E-05 *
Q5JU21	FPGS	Folylpolyglutamate synthase	0.62	6.92E-03 *	0.28	2.27E-02 *
P06132	UROD	Uroporphyrinogen decarboxylase	0.63	3.97E-04 *	0.70	1.53E-03 *
E9PGM4	GBE1	1,4-alpha-glucan-branching enzyme	0.63	4.41E-05 *	0.94	3.51E-02 *
Q6ZRI6	C1orf57	Uncharacterized protein C15orf39	0.64	2.87E-02 *	0.90	2.68E-01
A0A024RB32	PTGES3	Prostaglandin E synthase 3	0.64	2.58E-03 *	0.58	9.06E-03 *
P17858	PFKL	ATP-dependent 6-phosphofructokinase	0.65	8.97E-05 *	0.51	7.45E-06 *
Q3T7C7	TRIT1	tRNA dimethylallyltransferase	0.65	1.11E-02 *	0.96	3.27E-01
A0A087X2I1	PSMC6	26S protease regulatory subunit 10B	0.66	9.08E-04 *	0.95	2.18E-01
Q6FHU0	PSMB8	Proteasome subunit beta type	0.66	2.15E-02 *	0.65	3.82E-03 *
ALDH18A1	ALDH18A1	Glutamate 5-kinase	0.66	7.33E-04 *	0.74	1.61E-02 *
Q01970	PLCB3	1-phosphatidylinositol 4,5-bisphosphate phosphodiesterase beta-3	0.66	3.43E-02 *	1.18	6.41E-02

Metabolic enzymes that are down regulated in RPMI-8226 BTZ/100 cells compared to WT cells with fold change (FC) < 0.67

* = p<0.05 based on Student's T-Test

Supplemental Table S3: Characteristics of multiple myeloma patients

Patient	Gender	Age (yr)	BTZ treatment	Remarks
1	Male	68	no	Sample at diagnosis
2	Male	46	yes	Initial response to BTZ/dexamethasone therapy, autologous stem cell transplantation, relapse after 2 years, second BTZ/dexamethasone therapy and tandem auto/allo-transplantation. Relapse with development of plasma cell leukemia when sample was taken.
3	Female	66	yes	Relapse after autologous STC, start BTZ/dexamethasone therapy, development of plasma cell leukemia after 4 months therapy when sample was taken (BTZ-refractory)
4	Female	68	no	MM with breast cancer metastasis; starting therapy with melphalan when sample was taken
5	Female	65	yes	Progressive disease during BTZ/dexamethasone therapy when sample was taken (BTZ-refractory)
6	Male	70	no	Sample taken at first relapse after melphalan/ dexamethasone/ lenalidomide therapy.
